# Embargoes against American Beef Products

**Published:** 1895-03

**Authors:** 


Our contemporary, the *American Veterinary Review*, must find itself in a very embarrassing position now that the truth has come out explaining the embargoes against American beef products, said to be because of the prevalence of contagious pleuro-pneumonia and Texas fever, when leading foreign veterinarians admit there was no basis for this action on these grounds. How could we send them any contagious pleuro-pneumonia when we have not had a single case, acute or chronic, for nearly three years. As for Texas fever, we have never had so little, thanks to the good offices and regulations of the Bureau of Animal Industry. Why did these countries not take this action against us when we had and admitted the presence of contagions pleuro-pneumonia, and when Texas fever was frequently breaking out among herds and on pasture grounds at or near our seaboard cities? Why will he justify these foreign countries in their action, and cite high professional scientific grounds for their attitude, when we now know that the whole movement has had its origin because of the competition of American food products. We can raise better beef and undersell them in their own markets, and to allay the clamor and opposition of their own people they propose to raise up a Chinese wall of protection against our beef products, and hide under a false cloak that charges us with sending them diseased animals. They even could not claim the protection of a tuberculosis fear, for by actual statistics they have not found among our cattle, except in rare instances, any evidences of tuberculosis, while the same statistics show among their own a very serious percentage. This can readily be accounted for from the fact that few cattle are shipped abroad except those which come from our Western ranges, where tuberculosis is hardly known, and in great measure from sections of our country where contagious pleuro-pneumonia never secured a foothold. They adopted these scientific reasons, because if they admitted the truth, they feared we would adopt retaliatory measures against products from their countries; but thanks to the honor of members of our fellow profession abroad, they were unwilling to be placed in a false attitude before the world.

